# Perspective from single‐cell sequencing: Is inflammation in acute ischemic stroke beneficial or detrimental?

**DOI:** 10.1111/cns.14510

**Published:** 2023-10-31

**Authors:** Xinpeng Deng, Ziliang Hu, Shengjun Zhou, Yiwen Wu, Menglin Fu, Chenhui Zhou, Jie Sun, Xiang Gao, Yi Huang

**Affiliations:** ^1^ Department of Neurosurgery The First Affiliated Hospital of Ningbo University Ningbo China; ^2^ Key Laboratory of Precision Medicine for Atherosclerotic Diseases of Zhejiang Province Ningbo China; ^3^ School of Economics and Management China University of Geosciences Wuhan China

**Keywords:** acute ischemic stroke, astrocytes, microglia, single‐cell RNA sequencing

## Abstract

**Background:**

Acute ischemic stroke (AIS) is a common cerebrovascular event associated with high incidence, disability, and poor prognosis. Studies have shown that various cell types, including microglia, astrocytes, oligodendrocytes, neurons, and neutrophils, play complex roles in the early stages of AIS and significantly affect its prognosis. Thus, a comprehensive understanding of the mechanisms of action of these cells will be beneficial for improving stroke prognosis. With the rapid development of single‐cell sequencing technology, researchers have explored the pathophysiological mechanisms underlying AIS at the single‐cell level.

**Method:**

We systematically summarize the latest research on single‐cell sequencing in AIS.

**Result:**

In this review, we summarize the phenotypes and functions of microglia, astrocytes, oligodendrocytes, neurons, neutrophils, monocytes, and lymphocytes, as well as their respective subtypes, at different time points following AIS. In particular, we focused on the crosstalk between microglia and astrocytes, oligodendrocytes, and neurons. Our findings reveal diverse and sometimes opposing roles within the same cell type, with the possibility of interconversion between different subclusters.

**Conclusion:**

This review offers a pioneering exploration of the functions of various glial cells and cell subclusters after AIS, shedding light on their regulatory mechanisms that facilitate the transformation of detrimental cell subclusters towards those that are beneficial for improving the prognosis of AIS. This approach has the potential to advance the discovery of new specific targets and the development of drugs, thus representing a significant breakthrough in addressing the challenges in AIS treatment.

## Graphical abstract

Single‐cell sequencing reveals complex interactions of brain cells after acute ischemic stroke. After acute ischemic stroke, brain cells include microglia, astrocytes, peripheral blood macrophages, OPCs, oligodendrocytes, neutrophils, Th1 cells, CD8+ cells, B cells, NK cells, Treg cells, etc., cross‐talk with each other to participate in the disease mechanism.

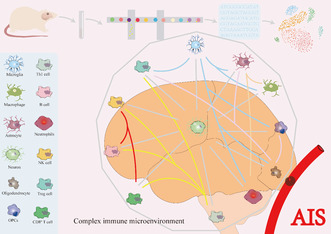

## 1 INTRODUCTION

Stroke is the leading cause of acquired disability worldwide and the second leading cause of dementia and death.^1^ In addition, stroke bears substantial significance in the realm of public health on a global level.^1^ AIS is the most common type of stroke, accounting for approximately 80% of all stroke cases. Globally, there are approximately 7.6 million new AIS cases each year, and some patients endure long‐term neurological and psychiatric disorders as a result.^1^, ^2^ AIS arises from the acute occlusion of the cervical or intracranial arteries, leading to a severe reduction in cerebral blood flow and the subsequent necrosis of the downstream brain tissue.^3^, ^4^ Currently, the main therapeutic strategy for ischemic stroke is to rapidly restore blood flow in the occluded vessel and reestablish the blood supply to the ischemic area via methods such as pharmacological thrombolysis and mechanical endovascular thrombectomy. However, reperfusion of the ischemic area can exacerbate brain injury, a phenomenon known as cerebral ischemia–reperfusion injury (CIRI).^5^ This process is complex and involves inflammation, apoptosis, oxidative damage, excitotoxicity, immune responses, and more.^6^, ^7^

Neuroinflammation and oxidative stress play significant roles in the diverse complex mechanisms of AIS.^8^ The activation of resident cells, including microglia, astrocytes, oligodendrocytes, and endothelial cells, following AIS can promote brain regeneration and recovery. However, these cells can also recruit immune cells expressing inflammatory mediators, causing disruption of the blood–brain barrier (BBB), neuronal death, brain edema, and hemorrhagic transformations.^9^ The regulation of inflammation and the promotion of the repair of vascular neuronal units are essential aspects influenced by the modulation of immune cells following stroke. Therefore, understanding this process is crucial for the advancement of stroke therapeutics.^9^, ^10^

Previous studies of ischemic stroke have often focused on bulk tissue mixtures, obscuring decisive changes in gene expression profiles within the most vulnerable cellular subpopulations.^11^ The application of single‐cell sequencing technology provides us with a good technical means to refine the classification of cell subclusters. Single cells are first isolated from tissue samples or cell samples through mechanical or enzymatic treatment to prepare single‐cell suspensions. Single cells are captured and aliquoted into individual reaction tubes using microfluidic chips, microfluidic tubes, or droplet‐based methods. The sequencing data was finally analyzed through bioinformatics to obtain the gene expression profile of a single cell.^12^, ^13^, ^14^, ^15^ With advancements in single‐cell technologies, research precision has reached the level of individual cells. Importantly, the acute inflammatory response in stroke patients involves the activation of resident brain cells and recruitment of peripheral immune cells. Thus, the proportion and characteristics of the infiltrating immune cells change at different time points.^16^ However, single‐cell RNA sequencing (scRNA‐seq) can reveal changes in gene expression within individual cells and precise transcriptional alterations during neuroinflammation. This provides a new approach for studying the heterogeneity among subpopulations of different cells, opening new avenues for exploring the pathogenesis of stroke and drug discovery based on cell subtype‐specific molecules.^2^

## 2 MICROGLIA

Previous studies have found a correlation between brain injury and elevated levels of reactive oxygen species (ROS) within cells.^17^ Brain cells are susceptible to hypoxia, which increases ROS levels. Additionally, during the process of ischemia/reperfusion (I/R), an excessive amount of ROS is generated, disrupting the antioxidant defense system and leading to oxidative stress, brain cell damage, and dysfunction of the BBB.^18^, ^19^ Furthermore, ROS‐mediated oxidative stress promotes neurodegeneration and demyelination in the ischemic areas, resulting in neuronal damage.

Following an ischemic brain injury, dramatic changes in the internal environment or ischemic neuronal death can activate microglial cells within minutes.^20^ As the predominant resident immune cells in the brain, microglial cells comprise the largest proportion of cells after cerebral I/R injury^21^ and are closely associated with the early excessive ROS response during I/R.^2^, ^22^, ^23^, ^24^, ^25^ The activation of microglial cells is the initial step in the central nervous system inflammatory response, followed by the infiltration of immune cells, such as neutrophils, macrophages/monocytes, natural killer cells, and T cells, and the activation of neurons.^26^ In a study by Zeng et al., the percentage of activated microglial cells was significantly higher than that in the sham‐operated group on the first, third, and seventh day after AIS.^21^ Moreover, activated microglial cells produce various mediators, including inducible nitric oxide synthase (iNOS), nitric oxide (NO),^27^, ^28^ pro‐inflammatory cytokines (such as TNF‐a), and anti‐inflammatory cytokines (such as TGF‐a),^29^ collectively leading to complex immune reactions in the brains of patients with stroke,^16^, ^30^, ^31^, ^32^ including reactions with contradictory effects.^33^, ^34^, ^35^ Furthermore, there are numerous receptor–ligand pairs between microglial and immune cells, indicating the central role of microglial cells in immune responses,^21^ as well as their critical involvement in neuroinflammatory‐related diseases.^36^ PRDX1 is a member of the peroxidase family that plays a crucial role in protecting cells from oxidative stress by detoxifying peroxides. Therefore, Kim et al. proposed that SAM are activated through a pathway dependent on PRDX1. Furthermore, a specific reduction in the number of SAM was observed in cases of PRDX1 loss, leading to exacerbation of ischemic injury. In comparison to *PRDX1+/+* mice, *PRDX1−/−* mice exhibited a significant increase in microglial cell death in the ipsilateral hemisphere, larger infarct volumes, and worse prognoses.^23^ Kim and colleagues primarily utilized markers such as *Cd68* and *Adgre1* to identify macrophages/monocytes, *Cxcr2* to designate granulocytes, *Cd3d* for lymphocytes, and *Tmem119*, *Cx3cr1*, and *Sall1* for microglial cells in their study. Their research revealed a significant reduction in the antioxidative function within prdx1‐deficient microglial cells, rendering them more susceptible to the accumulation of ROS and other oxidative byproducts.^23^ Ma et al.'s study also found that the inactivation of prdx1 would aggravate the oxidative stress of mitochondria,^37^ which to a certain extent indicates that the antioxidant effect of microglia may depend on prdx1.

**FIGURE 1:**
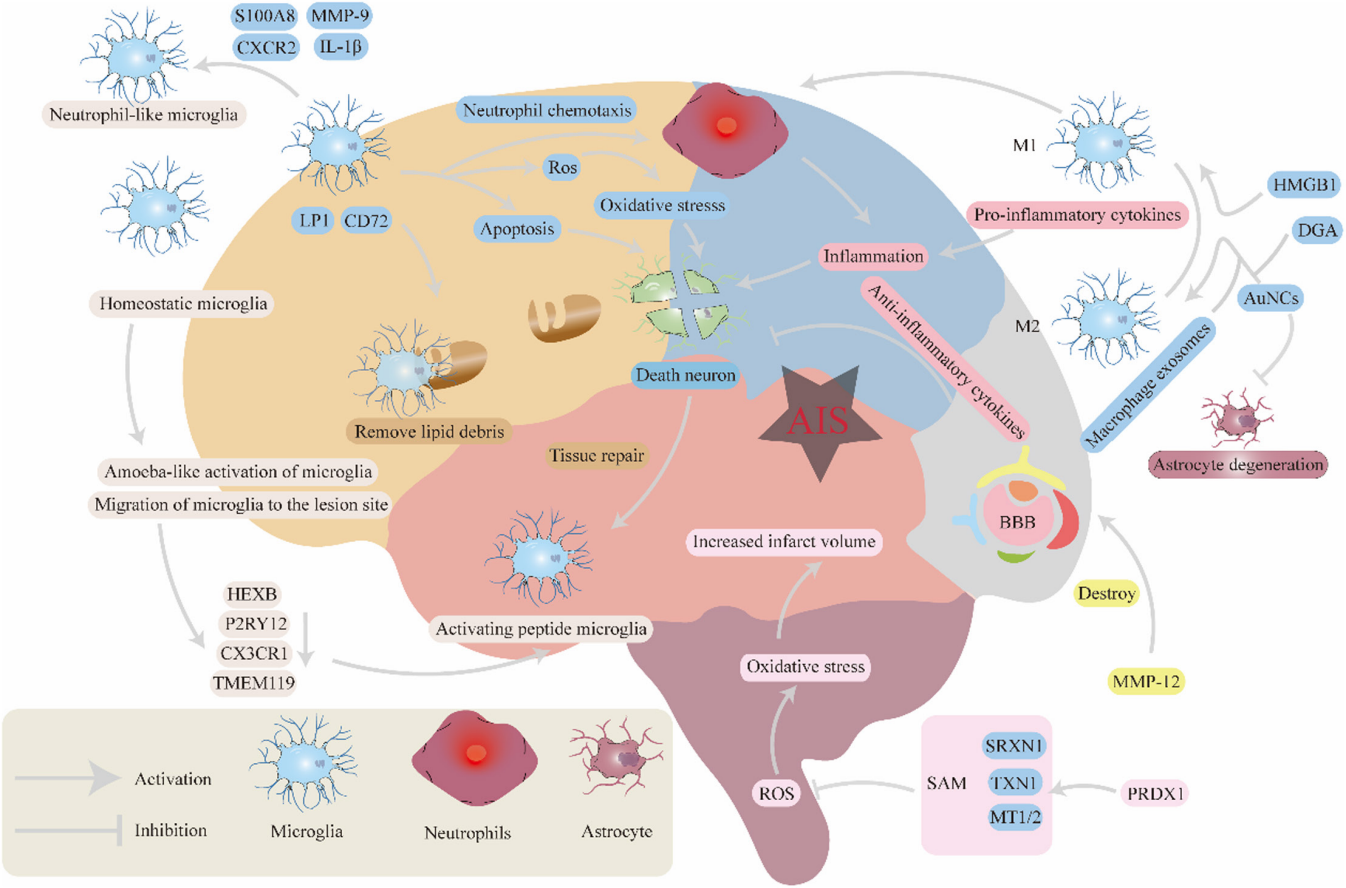
Key functions of microglia following AIS. AIS activates microglia through various factors, including ischemia, hypoxia, and neuronal death, subsequently leading to microglial proliferation. Notably, the M1 phenotype of microglia not only exacerbates neuronal damage through the secretion of pro‐inflammatory factors or the mediation of apoptotic pathways but also indirectly activates neutrophils, thereby intensifying the inflammatory response. The M2 phenotype of microglia can secrete anti‐inflammatory factors to mitigate brain injury.

Zheng et al. utilized scRNA‐seq to analyze cell populations in a mouse model of middle cerebral artery occlusion (MCAO) in the brain. Their study identified 17 major cell clusters, including smooth muscle cells (SMC), perivascular fibroblast‐like cells, central nervous system‐associated macrophages (CAM), monocyte‐derived cells (MdC), venous endothelial cells (vEC), capillary endothelial cells (capEC), arterial endothelial cells (aEC), pericytes (PC), choroidal plexus capillary endothelial cells (CPC), ependymocytes (EPC), microglial (MG) cells, neutrophils (NEUT), astrocytes (ACS), oligodendrocytes (OLG), neural progenitor cells (NPC), lymphocytes (LYM), and red blood cells (RBC).^2^ In total, 275 differentially expressed genes (DEGs) were identified between the MCAO and sham groups, with the highest number of DEGs observed in microglial cells. These DEGs in microglial cells were enriched in neutrophil chemotaxis and apoptosis signaling pathways. This suggests that microglial cells, which are intrinsic immune cells in the brain, play a crucial role in neuroinflammatory responses following ischemic stroke^2^ (Figure 1).

In their study, Zheng et al. made an intriguing discovery regarding the downregulation of the P2 purinergic receptor gene *P2RY12*, a microglial cell marker gene, in microglial cells after MCAO. This observation was consistent with previous reports showing decreased *P2RY12* expression in microglial cells upon inflammatory stimulation.^38^ This suggests that microglial cells may undergo a polarity shift or activation following AIS. Traditionally, microglial activation has been categorized into two types: classical (M1 type) and alternative (M2 type) activation. M1 microglial cells release pro‐inflammatory cytokines and neurotoxic mediators, leading to the exacerbation of neuronal injury. In contrast, M2 microglial cells facilitate repair and elicit anti‐inflammatory responses. After ischemic brain injury, microglial cells rapidly migrate to the lesion site and produce inflammatory cytokines and cytotoxic substances, thereby exacerbating tissue damage. However, on the other hand, microglial cells also promote tissue repair and remodeling through debris clearance and remodeling, including the production of anti‐inflammatory cytokines and growth factors.^39^ Specifically, LP1 and CD72 have been implicated in the clearance and recycling of lipid debris and regulation of microglial cell proliferation,^40^ and their specific upregulation in microglial cells after MCAO may be associated with tissue repair.^2^ Ultimately, understanding the detailed mechanisms of microglial activation and polarization will contribute to shifting microglial cells from harmful to protective phenotypes.^8^, ^41^

High‐mobility group box 1 (HMGB1) is a potent pro‐inflammatory mediator that promotes M1 polarization in microglial cells. In one study, Gao et al. found that the inhibition of HMGB1 could induce M2 polarization of microglial cells, thereby alleviating traumatic brain injury.^42^ Similarly, Jin et al. found that ROS‐responsive 18β‐glycyrrhetinic acid‐conjugated polymeric nanoparticles (DGA) manipulate microglial cell polarization by inhibiting the translocation of nuclear HMGB1, thereby suppressing M1 microglial cell activation, enhancing M2 microglial cell activation, inducing neuronal regeneration, reducing infarct volume, and restoring motor function.^43^ Additionally, AuNCs functionalized with dihydrolipoic acid (DHLA−AuNCs) can induce microglial cell polarization toward the M2 phenotype, effectively improving stroke‐related tissue damage and reducing astrocyte degeneration.^44^ Furthermore, it has been observed that macrophage‐derived exosomes polarize microglial cells from the M1 pro‐inflammatory phenotype to the M2 anti‐inflammatory phenotype. Moreover, as research has advanced, the simplistic M1/M2 dichotomy in microglial cell polarization has been recognized as an oversimplification.^45^

In terms of microglial cell diversity and subtypes, Zeng et al. identified eight clusters. Cluster 0 is characterized by high expression levels of *Neat1*, *Gm47283*, *Hspa1b*, and *Mki67*. Cluster 1 is distinguished by elevated expression of *Top2a* and *Gng11*. Research indicates that Top2a influences the progression of liver cancer by modulating the hepatic carcinoma immune microenvironment.^46^ Cluster 2 exhibits high expression of *Birc5*, *Cdk1*, and *Smc2*. Cluster 3 is marked by elevated expression of *Sparcl* and *Igfbp7*. Liu et al.'s study suggests that Igfbp7 can inhibit scar formation through the BRAF/MEK/ERK signaling pathway.^47^ There is reason to suspect that Cluster 3 may be involved in scar formation following AIS. Cluster 4 is characterized by high expression of *Cxcl2* and *Ube2c*. Cluster 5 shows high expression of *Ptn*. Cluster 6 demonstrates high expression of *Ly6c1*, *Cd83*, and *IL‐1α*. Cd83, as a crucial immune checkpoint, plays a pivotal role in the genesis of microglial cells.^48^ Lastly, Cluster 7 exhibits high expression of *Hist1h2ap* and *Lag3*. Cluster 1 is involved in inflammation, cluster 3 is associated with metabolic processes, and clusters 6 and 7 are involved in glial cell differentiation, gliogenesis, neurogenesis, and synapses.^21^ Zheng et al. also found that microglial cells exhibit cellular diversity in five different subtypes after stroke and divided these into five clusters. Cluster MG 0 primarily originates from the sham‐operated group and is characterized by high expression of genes such as *P2ry12*, *Selplg*, *Tmem119*, *Gpr34*, *Siglech*, *Olfm13*, *P2ry13*, *Csf1r*, and *Hexb*. Cluster MG 1 originates from both the sham‐operated and MCAO groups in equal proportions and exhibits high expression of genes including *Cx3cr1*, *Ccl12*, *Ler3*, *Ccl4*, and others. Cluster MG‐2 is closely related to inflammation and microglial infiltration, mainly characterized by high expression of *Spp1*, *Ccl4*, *Cd72*, and *Mmp12*.^49^ Neuroinflammation‐associated protein Spp1 was found to regulate the inflammatory response of microglia, cell metabolism of astrocytes, and iron death in ischemic stroke.^50^, ^51^ In addition, CCL4 plays an important role in oxidative stress,^52^ inducing inflammatory signaling and barrier breakdown of neurovascular endothelial cells.^53^ MG 3 displays high expression of genes such as *Usp18*, *Rsad2*, *Isg15*, *Ifit3*, *Irf7*, and *Cxcl10*, which are predominantly enriched in the interferon signaling pathway, suggesting a crucial role for the interferon signaling pathway in MG 3. MG 4 exhibits high expression of genes associated with proliferation, including *Birc5*, *Stmn1*, *Ube2c*, *Cdca8*, *Top2a*, and *Pdk*, suggesting that MG 4 is likely a reservoir of proliferating microglial cells. And MG 2, 3, and 4 originated from the MCAO group. Among them, MG 2, 3, and 4 were most relevant to AIS. Microglial cells from these five clusters expressed core markers *GPR34*, *OLFML3*, *P2RY12*, *TMEM119*, *SELPLG*, and *SIGLECH*, but their expression was relatively lower in the MG 2, 3, and 4 subclusters, which were associated with inflammatory ischemic injury.^2^ Similarly, Li et al. found in their study that steady‐state genes (such as *P2RY12*, *TMEM119*, *CX3CR1*, and *HEXB*) were expressed less in the MG 5 subcluster. MG5 mainly overexpresses *Tmsb4x, Actb, Cst3, mt‐Co3, Gm42418, C1qa, mt‐Atp6, Rps29, Ctsd*, and *C1qb*, which was most related to inflammation after stroke, compared to the resting state MG 1.^54^ Tmsb4x can promote the differentiation of peripheral blood monocytes into dendritic cells.^55^ Research by Yang et al. has proven that C1qa plays an important role in the regulation of the immune microenvironment of melanoma and is related to complement activation.^56^, ^57^ In MG5 cells, the expression of *Top2A*, *Stmn1*, *Mki67*, and *Cdk1* was significantly upregulated, indicating that MG5 cells were in a highly proliferative state. MG6 cells also express high levels of *Cxcr2*, *S100a8*, *Il1b*, and *Mmp9*, exhibiting a unique “neutrophil‐like” phenotype. One of the main characteristics of circulating neutrophils is elevated *CXCR2* marker.^58^ CXCR2 is associated with neutrophil recruitment in the tumor microenvironment.^59^ S100A8, as a neutrophil‐derived protein, has a strong correlation with neutrophils.^60^, ^61^ This new subtype may represent a stroke‐specific state of microglia in aged brains after stroke. MG6 genes are mainly enriched in immune pathways and cell adhesion signaling pathways.^54^ The study by Zhang et al. identified a relatively stable microglia subpopulation, MKI67+, CH25H+, and OASL+ microglia subpopulations. They found that MKI67+ microglia subclusters can proliferate and differentiate into CH25H+ and OASL+ subclusters. CH25H+ microglia exhibit enhanced phagocytic and neuroprotective properties after stroke. Infarct volume was significantly increased after ischemic stroke in Ch25h−/− mice compared with Ch25h+/−. At the same time, OASL+ subclusters accumulate in the ischemic brain, are related to the development of neuroinflammation after stroke, and are further aggravated in the brains of aged mice.^62^ This suggests that microglial cells undergo a transition from a steady state to an activated state following AIS to adapt to changes in their internal environment. Additionally, Zheng et al. found that as one of the most inflammatory ischemia‐related subgroups, the MG 2 subcluster shares a series of important genes with the microglial subclusters associated with neurodegeneration, including *LGALS3, LILRB4, LP1, SPP1*, and *FTH1*.^2^ The correlation of LGALS3 with inflammation has been described in many research areas. LILRB4 can regulate T‐cell suppression and infiltration.^63^ LGALS3 can mediate rheumatoid arthritis through the MAPK pathway.^64^ LGALS3‐transitioned inflammatory smooth muscle cells is a major regulator of atherosclerosis progression and inflammatory cell recruitment.^65^ Burbidge et al.'s study found that LGALS3 is associated with lysosomal membrane damage in the autophagy–lysosomal pathway in human midbrain dopamine neurons.^66^ This indicates that there may be common pathways or targets between AIS and neurodegenerative diseases and that these genes could be important breakthroughs in addressing these two disease categories.

In the aforementioned study by Zheng et al., it was observed that the MG 2 cluster exhibited high expression of matrix metalloproteinase 12 (MMP‐12). Previous studies have shown that MMP‐12 causes severe damage to the BBB after ischemic stroke,^67^ as well as that MMP‐12 knockdown can alleviate secondary brain injury after stroke.^68^ MMP‐12 can lead to blood–brain barrier disruption, inflammation, apoptosis, and demyelination after stroke. In animal models of ischemic stroke, the expression of MMP‐12 in the brain is significantly increased, and inhibition of MMP‐12 can reduce brain damage and promote the prognosis of neurological, sensorimotor, and cognitive functions. Therefore, MMP‐12 may be a potential target for the treatment of ischemic stroke.^68^, ^69^ Moreover, Wang et al. proposed that MMP‐12 could serve as a potential biomarker of ischemic stroke in obese patients.^70^ In addition to microglia, MG 6 cells express high levels of CXCR2, S100A8, Il1B, and MMP9, exhibiting a unique ‘neutrophil‐like phenotype.^54^

However, although microglia exhibit a rapid increase shortly after AIS, this increase is not unlimited. Kim et al. found that 24 h after AIS, there was a notable decrease in the proportion of microglia in the ipsilateral hemisphere of individuals with AIS, whereas the proportion of infiltrating macrophages and granulocytes improved.^23^ Furthermore, at the 48‐h mark after AIS, there was a significant decrease in microglia and a corresponding increase in infiltrating immune cells, particularly macrophages, in the ipsilateral hemisphere, reaching their peak levels.^23^ Consistent with this, a study by Ma et al. found that the proportion of microglia and oligodendrocytes decreased in the 24‐h TMCAO system compared to the 12‐h TMCAO system.^71^ However, the level of lymphocytes showed minimal change between 24 and 48 h, which is also consistent with previous studies^72^, ^73^ and suggests that lymphocytes may not play a dominant role in the early stage of AIS. After 48 h, the number of infiltrating immune cells gradually increased in the contralateral cerebral hemisphere compared to 24 h, indicating that the effects of ischemic injury spread to the contralateral cerebral hemisphere over time.^23^

Forty‐eight hours after AIS in mice, Kim et al. divided their microglia into two distinct clusters that responded differently to stroke I/R injury. In the ipsilateral cerebral hemisphere of the ischemic injury, the number of microglia in cluster 2 markedly increased. Conversely, both the total number of microglia and the number of microglial cluster 1 cells were decreased in the AIS models. Further analysis revealed a temporal transcriptome shift from microglial cluster 1 to cluster 2, with the microglial transcriptome pattern shifting from cluster 1 to cluster 2. To explain this result, Kim et al. hypothesized that microglial cluster 2, a distinctive microglial population associated with stroke injury, might possess a protective function against ischemic injury.^23^ Consequently, Kim et al. classified cluster 1 and cluster 2 microglia into homeostatic and stroke‐associated microglia (SAM), respectively. Notably, SAM demonstrated unique gene expression patterns that differed from those of homeostatic microglia, indicating a distinct regulatory profile. Moreover, genes upregulated in SAM were associated with antioxidant and oxygen‐binding pathways, and inflammatory pathways were associated with the ischemic injury phenotype. Specifically, significantly elevated expression levels of peroxiredoxin 1 (PRDX1), thioredoxin 1 (SRXN1), thioredoxin 1 (TXN1), and metallothionein 1 and 2 (MT1 and MT2) were observed in SAM. Previous studies have found that disease‐associated microglia (DAM), a new type of microglia associated with Alzheimer's disease and amyotrophic lateral sclerosis, contribute to limiting neurodegeneration.^74^ Experimental data showed that CX3CR1, TMEM119, P2RY12, SERINC3, SELPLG, and MARCKS, which are generally downregulated in DAM, were also downregulated in SAM, whereas SPP1, CD9, CD63, FTH1, LPl, CSTb, CST7, and CTSl, which are upregulated in DAM, were also upregulated in SAM. Interestingly, no significant enrichment of oxidative stress‐related genes was detected in the DAM group. While SAM and DAM share similar markers, SAM cells distinguish themselves by exhibiting abundant antioxidant properties, suggesting a unique protective role in the context of stroke injury. Interestingly, SAM represent a unique microglial population induced by stroke and can be induced by reducing ROS to limit oxidative stress.^23^

## 3 ASTROCYTES

Research has indicated that various cellular characteristics undergo rapid changes within the short 12‐h period following AIS. Astrocytes after 12 h were divided into AST12‐A, AST12‐B, and AST12‐C. Among them, AST12‐A mainly expresses *Gm26917*, *Gria2*, *Syne*, *Prisr*, *Nwdl*, *Tnil*, *Dpx17*, *Gm9925*, *Utp14b*, and *S1co1c1*. AST12‐B mainly expresses *Btbd17*, *Fzd2*, *Paqr7*, *Al464131*, *Dbp*, *Entpd2*, *Ppp1r3g*, *Gpr146*, and *Adora 2b*. AST12‐C mainly expresses *Plp1*, *Ptgds*, *Gfap*, *Mal*, *Mbp*, *Cldn11*, *Apod*, *Hspb1*, *S100a10*, and *Mobp*. The study by Ma et al. showed that the astrocytes of 24‐h TMCAO were significantly lower than those of 12‐h TMACO.^71^ *Agt, Itih3, ptch1, Slc6a9*, and *Spon1* were highly expressed in AST24_A. *DBP, ITM2A, SPOCK2, HIST2H2AA1*, and *RPS27RT* were highly expressed in AST24_B, whereas *CCl4, SPP1, CD14, GFAP*, and *CCI12* were highly expressed in AST24_C. Compared with AST24_B, AST24_C showed a marked increase in the expression of *SPP1, CCl2, CCl4, GFAP*, and *CD14*, whereas the expression of *ITEM2A* and *DBP* was significantly downregulated. Compared to astrocytes at 24 h, mitochondrial genes and oxidative phosphorylation pathways were upregulated at 12 h, which may indicate an increased energy demand, a greater degree of severe energy metabolism dysfunction, or a higher proportion of cells experiencing cell death. Moreover, further analysis revealed functional energy impairment in astrocytes within the 12‐h TMCAO system. This condition was reversed in the 24‐h TMCAO system, suggesting that astrocytes may have recovered from the state of energy functional impairment.

Previous studies have demonstrated the neuroprotective role of lactate after cerebral ischemia, which can improve nervous system outcomes.^75^, ^76^, ^77^ Ma et al. found that key enzymes related to glycolysis and glucose transporters were markedly reduced at 12 h, indicating a decrease in lactate production in astrocytes during this stage. Indeed, insufficient lactate levels may exacerbate neuronal energy deficiency after reperfusion, leading to adverse outcomes in AIS.^71^ Furthermore, gap junction and tight junction‐associated pathways were activated at 12 h, suggesting activation of the BBB and intercellular connections. However, this activation was reduced at 24 h, possibly indicating a weakened protective effect of the BBB, allowing more peripheral immune cells to enter the infarcted area. Moreover, AQP4 was upregulated at 12 h but significantly decreased at 24 h, suggesting a self‐protective role of astrocytes after ischemia, which is potentially beneficial in inhibiting the formation of brain edema.

At the 24‐h mark, astrocytes exhibit a greater tendency to function as signal amplifiers by releasing inflammatory signals, such as cytokines, in order to attract assistance from other cells.^71^ Notably, the 24‐h TMCAO system activated more inflammatory pathways, including cytokine–cytokine receptor interactions, chemokine signaling pathways, TNF signaling pathways, NF‐κB, and IL‐17 signaling pathways. Additionally, lysosomal, antigen processing, and presentation pathways were markedly upregulated at 24 h, indicating enhanced interactions between astrocytes and their microenvironment. Furthermore, the HIF‐1 and AGE‐RAGE signaling pathways were activated not only in the 12‐h TMCAO system but also in the 24‐h TMCAO system, suggesting that astrocytes in these two states are not completely distinct and share certain characteristics. It is worth mentioning that Zheng et al. found that the expression of GFAP, which is primarily upregulated in reactive astrocyte proliferation, is a significant marker. Consequently, excessive proliferation of reactive astrocytes can not only lead to scar formation, limiting inflammation but also restricting neuronal reconnection.^2^, ^78^

## 4 OLIGODENDROCYTES

Oligodendrocytes produce lipid‐rich membranes and myelin sheaths around axon bundles to support axonal signaling.^79^, ^80^, ^81^ However, it is increasingly recognized that in addition to myelin sheath formation and maintenance, different subpopulations of oligodendrocytes play different roles.^81^, ^82^ For instance, reactive oligodendrocyte hyperplasia can be observed during ischemic brain injury in preterm infants.^83^ Moreover, studies have shown that MCAO can induce a subpopulation of oligodendrocytes with a reactive phenotype and that oligodendrocytes repair white matter damage in response to ischemia.^84^

Zheng et al. found that oligodendrocytes are enriched in the pathway regulating neuronal apoptosis,^2^ which indicates that oligodendrocytes play a role in regulating neuronal homeostasis. Moreover, Frazier et al. found that oligodendrocyte responses to stroke depend on age.^84^ In juvenile mice at 25 days, oligodendrocyte progenitor cells (OPCs) were significantly increased in the injured striatum 3–7 days after MCAO, and mature oligodendrocytes were resistant to ischemia in the same time frame, contributing to the relative preservation of the subcortical white matter.^85^ In contrast, adult mice showed a loss of oligodendrocyte progenitors and mature oligodendrocytes during injury, resulting in more severe white matter damage. These data suggest that oligodendrocyte responsiveness to ischemic stroke is age‐dependent.^84^ Furthermore, 14 days after MCAO, the number of immature (Olig2+ EdU+ CC1‐) oligodendrocytes significantly increased in the ipsilateral striatum. However, from 14 to 28 days post‐MCAO, the density of immature oligodendrocytes decreased significantly, while the density of mature (CC1+) oligodendrocytes remained unchanged. This suggests that immature oligodendrocytes were unable to survive or mature during this period.

Frazier et al. divided the above oligodendrocyte population according to the following characteristics: oligodendrocyte precursor cells (OPC) (cell cluster 1), Cell cluster 1 mainly expresses *Junb*, *Socs3*, *Cldn11*, *Actb*, *Rpl38*, *Rps28*, *Mbnl2*, and *Marcksl1*, committed precursor cells (COP) (cell cluster 2), Cell cluster 2 mainly expresses *Cldn11*, *Eef2*, *Gm10076*, and *Pus1*, pre‐oligodendrocytes 1 (pre‐OL1) (cell cluster 3), Cell cluster 3 mainly expresses *Junb*, *Marcksl1*, *Eef2*, *Gm10076*, *Cdk4*, *Gstp1*, *Rplp0*, and *Col11a1*, pre‐oligodendrocytes 2 (pre‐OL2) (cell cluster 4), disease‐associated oligodendrocytes (DOLs) (cell cluster 5), myelin‐forming oligodendrocytes (MFOLs) (cell cluster 6), and mature oligodendrocytes (MOL) (cell cluster 7). Cell cluster 6 mainly expresses *Ptgds*, *ApoE*, *Ifi27*, *Gstp1*, *Neat1*, *Trf*, *Ptms*, and *Sez612*. Cell cluster 7 mainly expresses *Junb*, *Socs3*, *Rps28*, *Cebpd*, *Mbp*, *Ifi27*, *Rnf122*, *Mt2*, *B2m*, and *Mt1*. Notably, cell cluster 5 was predominantly present in the striatum ipsilateral to the MCAO. This DOL cluster expressed many immune inflammation‐related genes, including *MHCI* genes, *IL33, SERPIN3N, C4B*, and *KIK6*, which are thought to promote inflammation by cleaving protease‐activating receptors 1 and 2.^86^ Importantly, SERPIN3N, which encodes an anti‐chymotrypsin, is upregulated during neuroinflammation.^87^

The oligodendrocyte precursor genes *PDGFRA* and *CSPG4* were highly expressed in clusters 1 and 2, whereas increased *BCAS1* and *TSN3* expression was observed in cells from clusters 3 and 4. Meanwhile, clusters 6 and 7 expressed the more mature oligodendrocyte gene *PLP1*, and *BCAS1* was overexpressed in the ipsilateral MFOL and MOL populations. Notably, BCAS1 expression is thought to define newly differentiated myelinating cells in the adult brain.^88^ All these indications suggest that cell clusters 6 and 7 may be more inclined to form mature oligodendrocytes. Furthermore, cluster 6 demonstrated increased enrichment of ontogenetic pathways involved in active myelination; however, these pathways were not significant in cluster 7.

Oligodendrocytes are generally considered to be mature, nonproliferating, and terminally differentiated cells. However, in recent decades, increasing evidence has suggested that the fate of oligodendrocytes is more flexible.^89^, ^90^, ^91^ For example, Bai et al. observed the transformation of oligodendrocytes into astrocytes during the AO cell stage following acute brain injury.^92^ Moreover, it has also been observed that IL‐6 induces oligodendrocytes to form AO cells, and LIF and BMP4 may subsequently affect astrocyte‐specific differentiation.^93^, ^94^, ^95^, ^96^, ^97^

## 5 NEURONS

Neurons are the main executors of neural system functions. Ischemic stroke leads to irreversible neuronal damage, and neuronal survival is crucial for brain function recovery.^98^, ^99^, ^100^ Gao et al. analyzed gene expression in neurons using single‐cell sequencing technology, identifying 1264 upregulated DEGs and 573 downregulated DEGs in neurons after MCAO. Following MCAO, the expression levels of *HSPB1, SPP1, MT2A, GFAP, IFITM3, VIM, CRIP1*, and *GPD1* significantly increased.

Previous studies have shown that HSPB1 overexpression enhances the expression of pro‐inflammatory cytokines and activates glial cells without increasing neuronal apoptosis, thereby regulating acute neuroinflammation.^101^ Indeed, high expression of HSPB1 reduces brain infarction size and iron accumulation in rats, decreases ferritin levels, attenuates cell apoptosis, and increases the levels of antioxidant systems such as GPX4.^102^ Furthermore, the knockdown of VIM reduces hippocampal neuronal injury and cell apoptosis.^103^ These experimental data indicate that both protective and destructive factors were upregulated in neurons following AIS. Consequently, enhancing the upregulation of favorable genes and suppressing the upregulation of harmful genes have become breakthroughs in protecting neurons in AIS. Notably, Dengzhan Shengmai (DZSM) capsules, which are included in the Chinese Pharmacopeia 2015, have been widely used to treat ischemic stroke.^104^ After treatment with DZSM, a significant reduction in the expression of VIM and IFITM3 in neurons has been observed, highlighting these as potential therapeutic targets of DZSM.^105^

## 6 MONOCYTES

Monocytes recruited from the bloodstream toward inflammatory tissues after an ischemic stroke play an important role in wound healing and tissue repair, representing an important immune response. The study by Xie et al. found that peripheral monocyte subsets were significantly increased after I/R. Cathepsin S (Ctss) is a key molecule that regulates monocyte activation, and its expression is highest 3 days after I/R. After knocking out *Ctss*, the infarct size, cell apoptosis, blood–brain barrier damage rate, and vascular leakage rate were significantly reduced.^106^ In mice, the monocytes can be divided into two distinct populations: pro‐inflammatory monocytes expressing CCR2highCX3CR1low, which circulate in the blood, and anti‐inflammatory monocytes expressing CCR2lowCX3CR1high, which patrol locally.^107^ Park et al. found that CCR2highCX3CR1low monocytes recruited to the injured brain undergo cell‐dependent conversion to CCR2lowCX3CR1high monocytes, particularly under the influence of IL‐13, thereby alleviating neuroinflammation after ischemic stroke. Thus, modulation of monocyte conversion is an important repair strategy for ischemic stroke.^107^ Furthermore, the conversion of CCR2highCX3CR1low monocytes in the damaged brain tissue may be part of the anti‐inflammatory response, contributing to maintaining homeostasis of the microenvironment after ischemic stroke. Therefore, the discovery of monocyte conversion in inflammatory tissues has provided new insights into ischemic stroke.^107^

CD14+ monocytes can be classified into two groups: dendritic cell‐associated CD14+ monocytes and NK cell‐associated CD14+ monocytes. In patients with stroke, Cho et al. observed a significant reduction in CD14+ monocyte subpopulations.^108^ Similarly, Zheng et al. observed a significant increase in the number of infiltrating monocyte‐derived cells (MdCs) in the MCAO group, with the cell proportion increasing from 2% to 16%. These experimental results indicate that even within monocytes, there are diverse directional changes between different subsets.

Recruitment of MdCs to the ischemic hemisphere may be closely related to key functions of innate immunity, cell adhesion, and phagocytosis in acute ischemic injury.^2^, ^109^ Li et al. identified five monocyte/macrophage clusters (MM1–5). Among them, the MM1 and MM2 clusters exhibited high expression of CCR2, FN1, and CYBB, representing the major monocyte/macrophage populations in the brain after AIS.^54^ CCR2 is a key chemokine ligand for inflammation that can trigger mast cell degranulation and activate inflammation.^110^ Based on the previous monocyte dichotomy, high CCR2 expression may indicate a pro‐inflammatory monocyte cluster, potentially playing a negative pro‐inflammatory role in the inflammatory cascade response of AIS and exacerbating brain damage.^107^

Peripheral immune cells are considered as potential biomarkers of ischemic stroke.^111^, ^112^ Among the circulating immune cells, monocytes have been investigated as potential biomarkers. Studies have revealed that each monocyte subtype is correlated with stroke severity or infections linked to stroke.^113^

## 7 NEUTROPHILS

During a stroke episode, neutrophils are rapidly mobilized from the periphery, cross the BBB, and reach the ischemic brain parenchyma, reaching their peak 24 h after ischemic stroke.^114^ As the first peripheral immune cells enter the brain after ischemic stroke, neutrophils are believed to contribute to the disruption of the BBB, edema, and oxidative stress, which are important factors in postischemic brain injury.^115^ Huang et al. found that neutrophil recruitment is associated with microvascular occlusion, which may exacerbate poststroke brain damage. This process is regulated by microglia and astrocytes. Microglia suppress astrocytic CXCL1 expression by secreting IL‐1RA, ultimately reversing the damage caused by neutrophil recruitment.^115^ In the early stages of cerebral ischemia, a higher neutrophil count in the peripheral blood is associated with a larger infarct volume, poorer clinical symptoms, and worse prognosis.^116^, ^117^ As a marker of systemic inflammation, the neutrophil‐to‐lymphocyte ratio (NLR) is an excellent predictive indicator of a series of events following ischemic stroke.^118^

Further research has shown that neutrophils play a dual role in AIS by exerting a protective effect and exacerbating brain injury. This functional duality is based on two different functional phenotypes of neutrophils, N1 and N2. The N1 phenotype promotes inflammation and worsens brain damage, whereas the N2 phenotype inhibits inflammation and promotes neurorepair. Neutrophils polarize into the N1 or N2 phenotypes after ischemic stroke.^119^, ^120^ In the early stages of AIS, N1 neutrophils exacerbate brain damage by overexpressing pro‐inflammatory factors, proteases, and ROS. Later, N2 neutrophils gradually dominate and exert neuroprotective effects by overexpressing cytokines.^121^ The enhanced phagocytic ability of N2 neutrophils has been observed in studies on AIS, leading to accelerated clearance of inflammatory tissue debris and promotion of tissue repair. Additionally, N2 neutrophils are preferentially engulfed by microglia and macrophages, which facilitates their clearance. The coordinated interaction between neutrophils and microglia forms an immune defense line after cerebral infarction and has potential neuroprotective effects.^122^, ^123^, ^124^, ^125^ Furthermore, the N1 and N2 phenotypes exhibit high plasticity and can undergo mutual conversion under certain conditions.^121^ Thus, regulating the mutual conversion of the N1 and N2 phenotypes is a promising therapeutic strategy for AIS.^119^, ^120^

**FIGURE 2:**
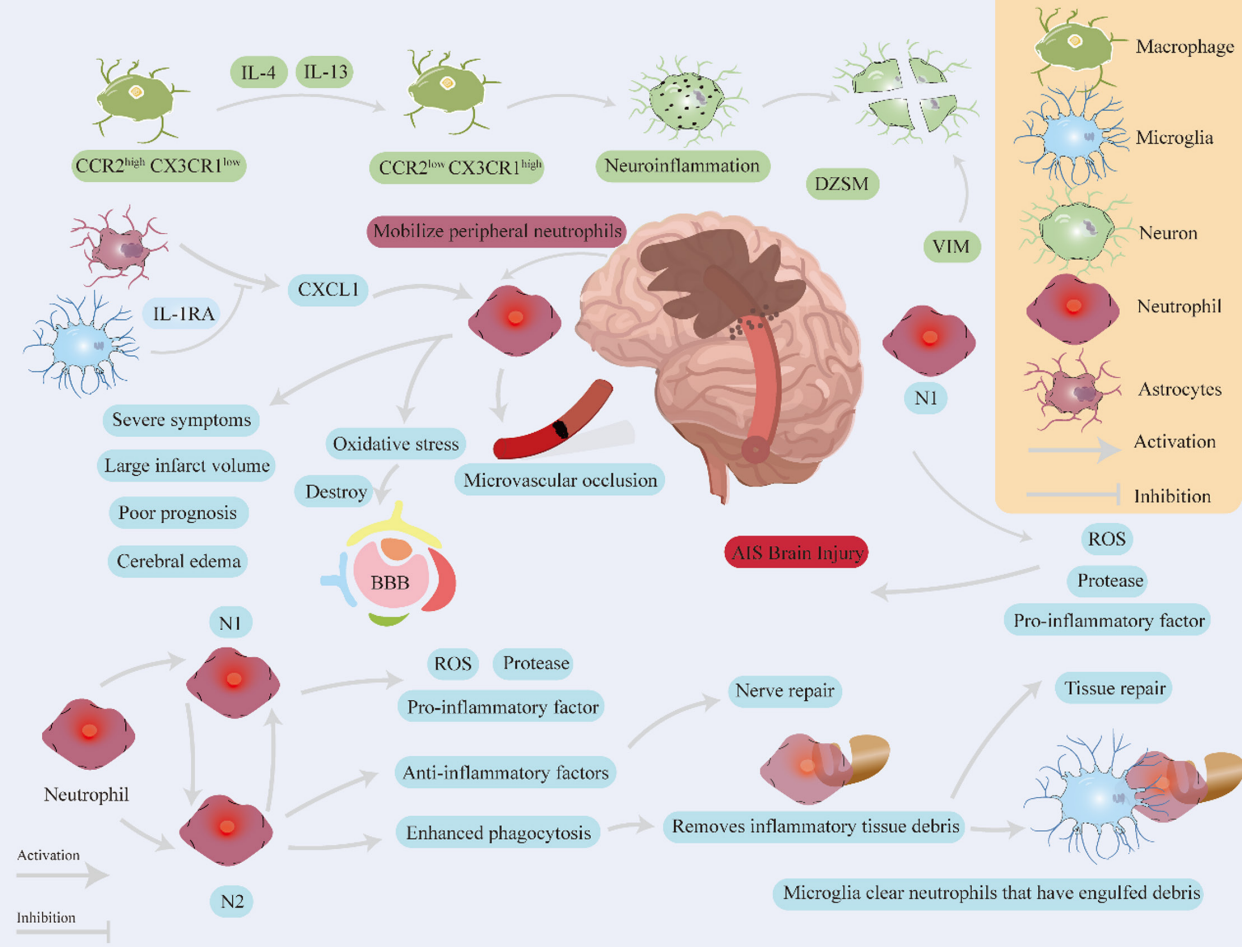
The role of neutrophils after the onset of AIS. Astrocytes can mediate the accumulation of neutrophils after AIS by regulating the production of CXCL1. Neutrophils are divided into two functional phenotypes: N1 and N2. N1 is a pro‐inflammatory phenotype, and N2 is an anti‐inflammatory phenotype. Manipulating the interconversion between these phenotypes may offer a novel avenue for potential breakthroughs.

With the development of single‐cell sequencing technology, it is increasingly recognized that neutrophils cannot be simply classified using a binary approach, and the true classification method is more complex. In their research, Zheng et al. identified four distinct neutrophil clusters: NEUT0 (PMNc‐G5c), NEUT1 (PMNb‐G5b‐ISG), NEUT2 (PMNa‐G5a), and NEUT3 (immune‐g2‐4).^2^ NEUT0 mainly expresses *Ccl3*, *Hcar2*, *Ccrl2*, *Bcl2a1a*, *Cd63*, *Ptafr*, *Gm5483*, *Cxcl1*, and *Ifitm1*. NEUT1 mainly expresses *Retnlg*, *Gbp2*, *Mmp8*, *Irf7*, *Hp*, *Isg15*, and *S100a8*. NEUT2 mainly expresses *IL‐1β*, *Slfn1*, *Cxcl2*, *Gm13822*, *Ltb4r1*, *Cfp*, and *Rdn12*. NEUT3 is mainly highly expressed in *Ngp*, *Camp*, *Ltf*, *Cd177*, *Chil3*, *Lyz2*, *Lcn2*, *Wfdc21*, *Cebpe*, *S100a9*, *Cybb*, and *Mmp9*. NEUT0 is mainly enriched in neutrophil degranulation and neutrophil activation involved in immune response, neutrophil mediated immunity, cellular response to interferon‐gamma, and regulation of adiponectin secretion. NEUT1 is mainly enriched in type I interferon signaling pathway, cellular response to type I interferon, antimicrobial humoral immune, response mediated by antimicrobia l peptide, interferon‐gamma‐mediated signaling pathway, and cellular response to interferon‐gamma. NEUT2 is mainly enriched in cellular response to cytokine stimulus, cytokine‐mediated signaling pathway, inflammatory response, negative regulation of insulin, receptor signaling pathway, and negative regulation of cellular response to insulin stimulus. NEUT3 is mainly enriched in granulocyte migration, defense response to fungus, innate immune response in mucosa, positive regulation of vesicle fusion, and neutrophil extravasation. Notably, NEUT0 significantly expressed much higher mobilization signals (Cxcl1), promoting the release of mature neutrophils from the bone marrow into the circulation, while also upregulating CD63, PTAFR, and HCAR2, which are associated with neutrophil‐mediated immune responses.^126^ In contrast, NEUT1 was characterized by a high expression of IFN‐related genes, including *IFITM1, GBP2, ISG15*, and *IRF7*. Meanwhile, NEUT2 cells exhibited high expression of *STFA2l1, CXCR2*, and *LTB4R1*, as well as genes associated with cellular responses to cytokine stimulation (*TREM1, FPR1*, and *CCR1*).^2^ In another study by Li et al., three neutrophil subsets were identified, with the primary NEU1 and NEU2 subsets showing high levels of *CD45*, *ITGAM* (*CD11B*), and CXCR2. Overall, stroke profoundly alters the immune system of the brain^54^ (Figure 2).

## 8 LYMPHOCYTES

**FIGURE 3:**
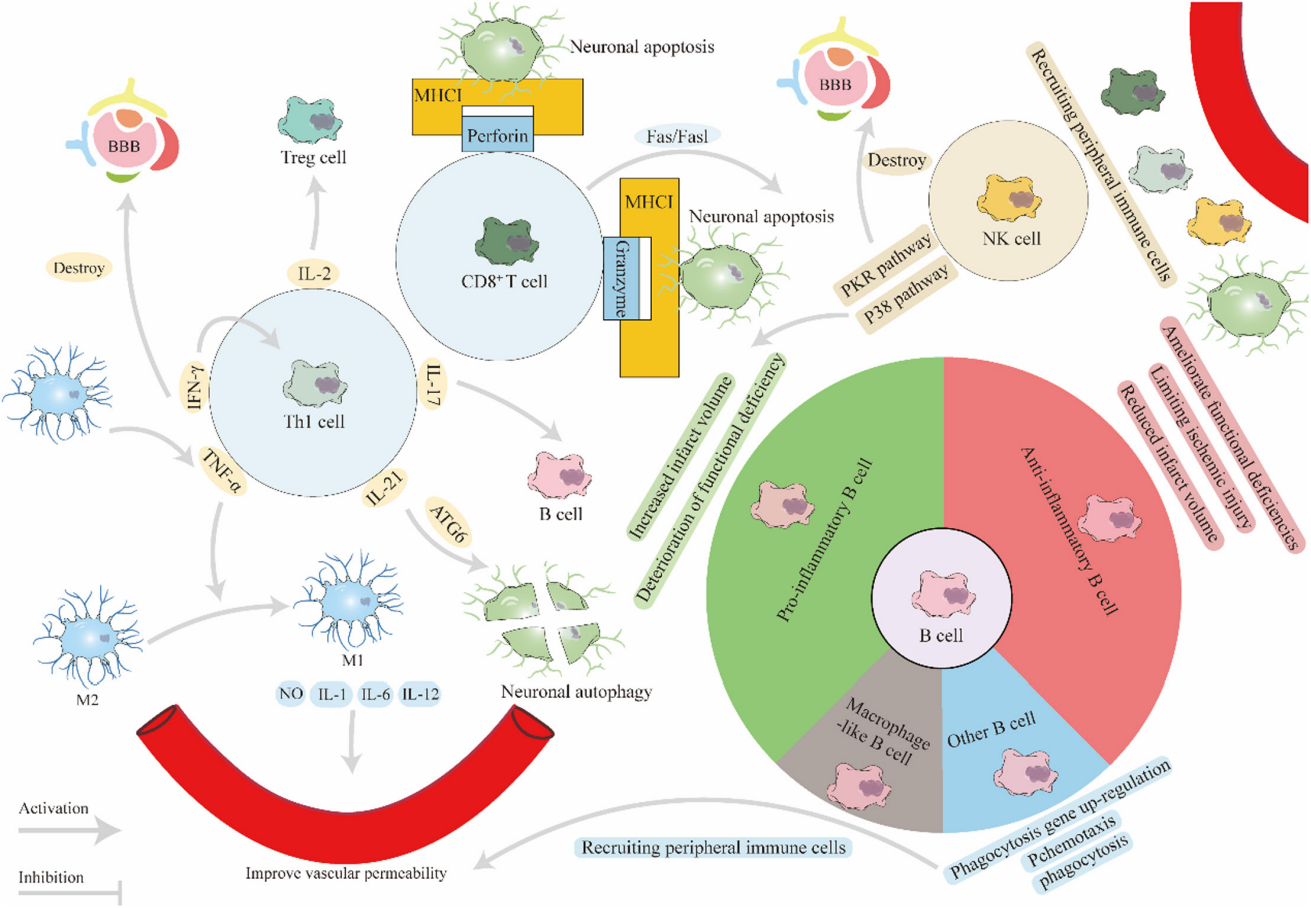
Th1 lymphocytes among CD4+ lymphocytes can secrete various cytokines and cross talk with microglia, neurons, Treg cells, and B cells, destroying the blood–brain barrier and aggravating brain damage. CD8+ cells can bind to MHCI molecules on the surface of neurons through ligands, leading to neuronal apoptosis. B cells not only have pro‐inflammatory and anti‐inflammatory effects, but can also recruit peripheral immune cells, forming an extremely complex immune microenvironment after AIS.

Uncontrolled excessive inflammatory response exacerbates ischemic brain injury, impeding recovery after stroke. In AIS patients, some types of T cells can promote the development of inflammatory responses and aggravate ischemic injury, while other T cells appear to exert neuroprotective effects through mechanisms such as immunosuppression.^127^ There are many subtypes of T cells, and different subtypes exhibit different functions (Figure 3). The main ones are CD4+ T cells and CD8+ T cells, as well as natural killer T cells (NKT) and regulatory T cells (Tregs).^128^, ^129^, ^130^ CD4+ T cells infiltrated the infarct area on the first day and increased significantly thereafter, reaching a peak on the seventh day. Seven days after AIS, some subtypes of CD4+ T cells continued to infiltrate the infarct area.^131^, ^132^ Among the various subtypes of CD4+ T cells, the number of helper T cells 1 (Th1) and Th17 cells increased in the first 3 days and decreased on the seventh day, while the trend of Th2 cells was exactly the opposite.^133^ CD4 + ‐mediated stroke damage is mainly caused by the pro‐inflammatory effect of Th1 cells. Th1 cells mainly produce a variety of pro‐inflammatory cytokines (IL‐2, IL‐21, TNF‐α, IFN‐γ, etc.), promote immune cell infiltration, activate inflammatory responses, and thereby cause tissue damage. Th1 cells secrete IL‐2 to promote the activation of T cells, B cells, macrophages, and induce the toxicity of CD8+ T cells; at the same time, it also promotes the activation of Treg.^130^ IL‐21 interacts with the IL‐21 receptor on neurons to upregulate the mRNA level of autophagy‐related gene 6 (*ATG6*), induce autophagy, and aggravate tissue damage.^134^ TNF‐α participates in various inflammatory reactions in the body and exerts activation and chemotactic effects on neutrophils. TNF‐α also promotes the transformation of microglia into a pro‐inflammatory phenotype, leading to the release of pro‐inflammatory mediators such as IL‐1, IL‐6, IL‐12, and nitric oxide (NO), potentially increasing vascular permeability.^135^ IFN‐γ disrupts the blood–brain barrier connection and promotes Th1 infiltration by activating IFN‐γ‐induced proteins.^136^ In addition, IFN‐γ can activate macrophages and enhance the secretion of cytokines such as TNF‐α.^137^ CD4+ cells also play an important role in B‐cell infiltration after stroke. IL‐17 secreted by CD4+ and various CCL and CXCL family members together guide myeloid cells to surround T cells and B cells, forming clusters and ectopic lymphoid structures, thereby promoting the expansion and differentiation of B cells.^138^ Experiments have shown that reducing CD4+ cell infiltration can reduce B‐cell infiltration. CD4+ cells mainly exacerbate the progression of stroke by secreting cytokines that promote inflammatory responses. Some studies have also shown that CD4+ cells can directly induce nerve cell apoptosis through a fas‐dependent mechanism.^139^

CD8+ T cells infiltrate into brain tissue within hours after stroke and are the first batch of invading lymphocytes, which increase significantly within 3 days after stroke.^132^ In AIS, MHCI‐like (MHCI) molecules appear on the surface of nerve cells under the influence of pro‐inflammatory factors such as IFN‐γ and TNF‐α. CD8+ can recognize MHCI and induce neuronal apoptosis by secreting cytotoxic granules containing perforin and granzymes or through TNF family receptor‐mediated cytotoxicity, and can also mediate cytotoxic effects through the Fas/FasL signaling pathway.^140^, ^141^ The increased number of CD8+ cells after AIS may increase the risk of complications such as cerebral hemorrhage.^127^, ^142^ The accumulation of CD8+ cells more than 30 days after stroke will adversely affect the prognosis of later stroke.^140^

Some animal studies suggest that B cells have a protective role after stroke. After middle cerebral artery occlusion, the B‐cell‐deficient group was observed to have larger infarct size, higher mortality, and more severe functional deficits.^143^, ^144^, ^145^ Additionally, another study found that B‐cell deficiency did not affect infarct volume or functional outcomes, suggesting that B cells do not have a significant impact on infarct progression.^146^, ^147^ The conflicting results may be due to the heterogeneity of B‐cell subsets and/or various methods of B‐cell depletion. Recent studies have identified a small but potent subset of regulatory B cells that produce anti‐inflammatory cytokines, such as IL‐10, that can limit clinical symptoms of ischemic injury and neurological disease.^144^, ^148^ However, there are also some subpopulations of B cells that play a pro‐inflammatory role. A unique CD11bhigh B cell cluster expressing CD11b, a marker normally expressed on myeloid cells, was found to significantly accumulate in the brain and contribute to neuroinflammation in aging and after stroke.^149^ Wang et al. identified a novel phenotype of macrophage‐like B cells expressing high levels of CD45 in an animal model of ischemic stroke. Such cells may be induced by specific upregulation of CEBP family transcription factors to the myeloid system or downregulation of Pax5 transcription factors to the lymphatic system. Macrophage‐like B cells are characterized by co‐expression of B cell and macrophage markers. Compared with other B cells, macrophage‐like B cells had upregulated expression of phagocyte‐related genes, including phagosome and lysosome‐related genes. So these cells have stronger phagocytosis and chemotaxis. In addition, macrophage‐like B cells recruit peripheral immune cells by releasing a variety of chemokines mainly through the CCL pathway, leading to a complex immune microenvironment in ischemic stroke.^150^

Zheng et al. identified six lymphocyte subsets: NK1 cells (*KIRB1C* and *IFNG*), NK2 cells (*S100A1* and *CAR2*), B1 cells (*CD79A* and *LY6D*), B2 cells (*LMO4* and *RAMP1*), T1 cells (*CD3D*, *CD160*, and *MS4A6B*), and proliferating T cells (*STMN1*, *UBE2C*, and *BIRC5*).^2^ Furthermore, in stroke patients, Cho et al. observed an increase in CD4+ T cells and NK cells, enhanced cell activity, and decreased CD8+ T cells.^108^ Notably, elevated levels of circulating NK cells and low levels of interferon‐gamma (IFN‐γ) or low levels of NK cell perforin expression are associated with a higher risk of infection after stroke.^151^, ^152^ In a study by Cho et al., among the top 20 DEGs in the NK cell cluster, only two genes (*CX3CR1* and *GIMAP7*) were markedly upregulated in stroke patients, whereas 18 genes (*CXCR4, DUSP2*, and *IL7R*) were downregulated. Despite the overall enhanced activity, most DEGs were downregulated, suggesting the involvement of posttranscriptional or posttranslational modifications in the potential mechanisms.^108^

The top five representative pathways among all of the DEGs in the NK cells included the NK cell signaling pathway, PKR pathway in interferon induction, and p38 MAPK signaling pathway, indicating enhanced NK cell activity in stroke.^108^ As activated NK cells play a crucial role in defense against infections or transformed cells and mediate inflammatory responses through the release of cytokines and chemokines,^153^ the IFN‐p38 MAPK‐nuclear factor kappa B (NF‐κB) pathway is a major signaling pathway involved in NK cell activation. Moreover, in ischemic stroke, the NF‐κB signaling pathway is implicated in acute responses and plays a role in BBB disruption, inflammation, and neuronal cell death.^154^ In addition, NF‐κB activity is associated with the severity of stroke, and inhibition of NF‐κB has been shown to reduce the size of infarction.^155^ Thus, the findings of Cho et al. suggest that targeting the NF‐κB pathway in NK cells could be a more specific therapeutic approach for the treatment of mild ischemic stroke.

## 9 CROSS TALK BETWEEN GLIAL CELLS

As one of the largest groups of immune cells in the brain,^156^ microglia play a significant role in the development of AIS. Activated microglial cells in the penumbra engulf blood vessels by extending their processes, resulting in the extravasation of blood‐borne macrophages. In addition, perivascular microglial cells phagocytose endothelial cells, leading to endothelial dysfunction and BBB.^157^ The cytokines released by microglia cells, such as IL‐1a, TNF‐α, and complement component 1q (C1q), can induce A1‐reactive astrocytes.^78^, ^158^ Li et al. found that extracellular vesicles (sEVs) derived from M2 microglial cells after AIS inhibited the proliferation and migration of astrocytes, ultimately reducing glial scar formation and promoting functional recovery.^159^ Moreover, the secretion of IL‐1b can increase the secretion of CCL2, CCL20, and CXCL2 and downregulate the expression of SHH signaling, which is a protective signal in astrocytes, following ischemic stroke.^160^

A previous study demonstrated that glucagon‐like peptide‐1 receptor agonists can inhibit microglial activation and M1 polarization, effectively suppress the transformation of A1 astrocytes, and exert a neuroprotective function in Parkinson's disease.^161^ In addition, astrocytes upregulate the expression of CX3CR1 and IL4RA in microglial cells through TGF‐b, thereby inhibiting the excessive activation of microglial cells and reducing BBB damage after AIS.^162^ Furthermore, astrocytes facilitate the entry of infiltrating monocytes and macrophages through interactions between fractalkine and CX3CR1.^163^ Moreover, extracellular vesicles derived from macrophages polarize microglial cells from the M1 pro‐inflammatory phenotype to the M2 anti‐inflammatory phenotype, thus playing a protective role against AIS.^164^

**FIGURE 4:**
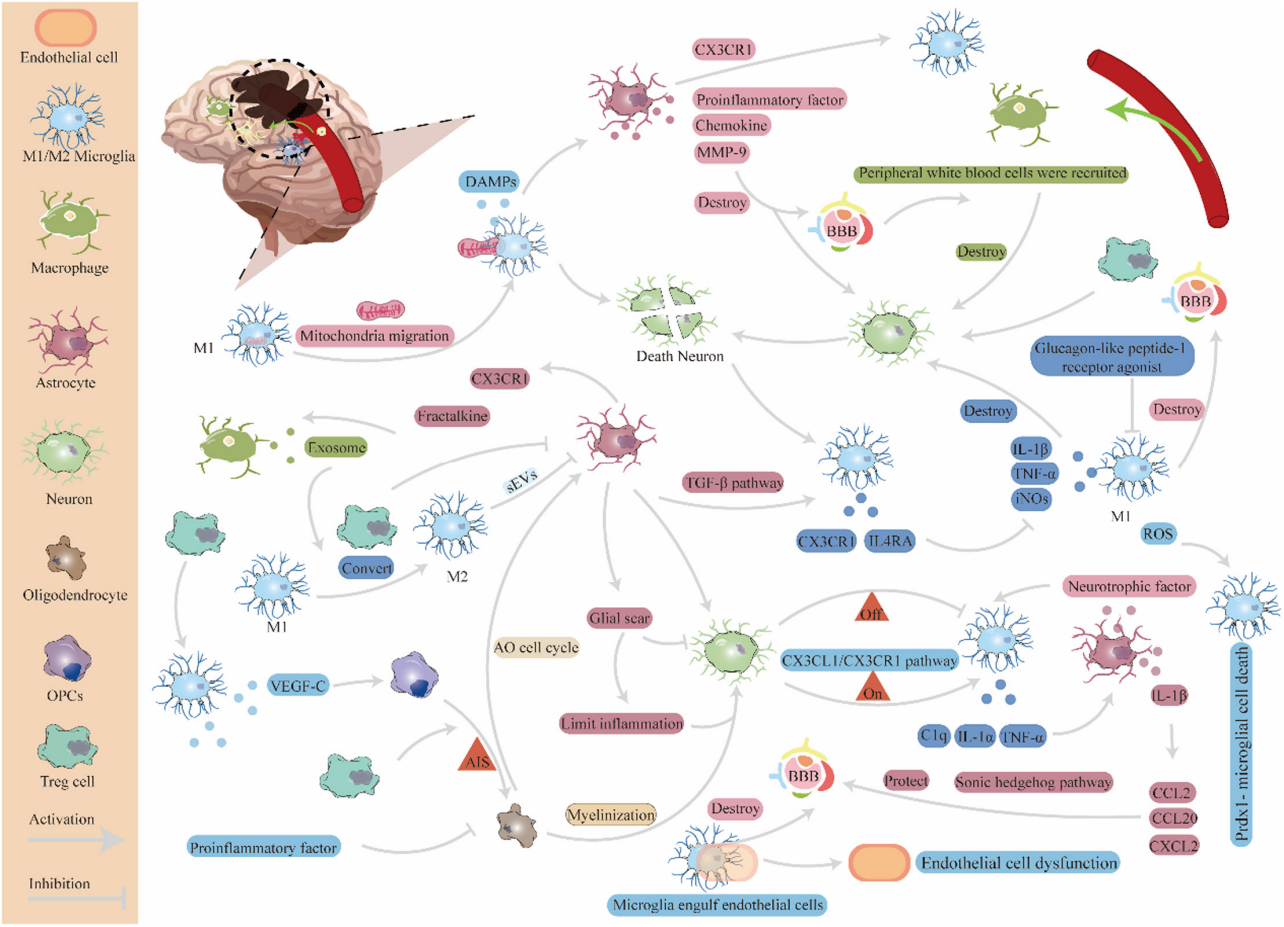
Cross talk in glial cells. The interaction between microglia and astrocytes, oligodendrocytes, and neurons is very complex, and it can develop toward the direction of reducing brain damage or aggravating brain damage. Microglia can activate astrocytes and lead to the formation of glial scars, and M2 microglia can also inhibit the proliferation and migration of astrocytes through sEVs, reduce the formation of glial scars, and promote functional recovery. In addition, microglia can also downregulate protective signaling in astrocytes, aggravating brain injury after AIS. Similarly, the roles of astrocytes vary. Astrocytes can upregulate the expression of CX3CR1 and IL4RA on the surface of microglia, inhibit the activation of microglia, alleviate brain damage, and indirectly induce polarization of the M1 pro‐inflammatory phenotype to the M2 anti‐inflammatory phenotype, exerting a protective effect in AIS. Neurons can also both upregulate microglia and downregulate glia through the CX3CL1/CX3CR1 signaling pathway. Furthermore, while M1 microglia aggravate the inflammatory response and lead to neuronal damage, M2 microglia can also exert neuroprotective effects.

Neurons and glial cells maintain homeostasis within the central nervous system through various intracellular and intercellular signaling mechanisms^165^ (Figure 4). After AIS, neurons are the main “victims.”^9^ Under pathological or physiological conditions, neurons are able to control the activation of microglia cells through “On” and “Off” signals released by neurons and their interaction with receptors on microglia cells.^166^, ^167^ Interestingly, neurons can both activate and inhibit the activation of microglia cells. Initially, microglial cell activation after ischemia is triggered by neuronal death.^168^, ^169^ In contrast, in the normal brain, neurons can inhibit the activation of microglial cells through the CX3CL1/CX3CR1 signaling pathway.^170^ In another study, Liu et al. discovered that activated microglial cells transformed into the pro‐inflammatory M1 type, which accelerated mitochondrial fission, leading to the release of damaged mitochondria. These mitochondria then further migrated into the neurons and fused with the neuronal mitochondria, resulting in impaired mitochondrial function, decreased ATP production, decreased mitochondrial membrane potential, increased ROS levels, and subsequent mitochondria‐mediated neuronal death, which worsens ischemic injury. Moreover, it was found that activation of M1 microglia leads to increased secretion of pro‐inflammatory cytokines, including IL‐1β, iNOS, and TNF‐β, causing a cytokine storm and accelerating neuronal damage.^171^ Similarly, neurons can affect the function of microglial cells, and damaged neurons after ischemia can stimulate microglial cells to exert neuroprotective effects.^166^

After AIS, astrocytes play a crucial role in supporting neuronal regeneration through the transportation of nutrients and lipids. Additionally, their reactive proliferation results in the formation of glial scars, which act as physical barriers, isolating the infarcted area and limiting the spread of inflammation.^172^, ^173^ However, glial scarring can hinder neuronal reconnection and extension.^9^ Neurons and glial cells at the site of injury are immediately activated by the release of damage‐associated molecular patterns and other products. Upon activation, reactive astrocytes release pro‐inflammatory cytokines, chemokines, and matrix metalloproteinases (such as MMP‐9), which can disrupt the BBB and attract leukocytes from the peripheral blood to the site of inflammation.^174^ This leads to secondary damage to the brain tissue. Additionally, astrocytes can secrete neurotrophic factors to protect the injured site.^174^

Oligodendrocytes provide myelin sheaths for axons^175^; however, the myelin phospholipid structure is typically disrupted after stroke,^176^ and OPCs, which give rise to mature oligodendrocytes, are vulnerable during ischemia.^177^ In stroke, microglia and macrophages (MG/MΦ) may drive angiogenesis and oligodendrogenesis through paracrine mechanisms.^178^ In this situation, the ability of OPCs to mature into oligodendrocytes is impaired, leading to failed remyelination and hindered neural recovery after stroke.^176^ In the past, studies have confirmed that the influence of microglial cells on OPCs plays a crucial role in poststroke remyelination. Inflammatory factors generated by activated microglia cells can have detrimental effects on oligodendrocytes and OPCs, impairing their function and viability.^166^, ^179^ In contrast, microglia cells produce vascular endothelial growth factor C (VEGF‐C) following ischemia, which stimulates the proliferation of OPCs via the VEGFR‐3 receptor.^180^ This finding suggests a dual role for microglia in the regulation of OPCs. Moreover, the transformation of M1 microglial cells into M2 microglial cells is related to remyelination. M2 microglia cells, as protective cells after stroke, can drive OPC differentiation, which is an essential component of an effective remyelination response.^181^

## 10 DISCUSSION

AIS is one of the most common cerebrovascular events worldwide and imposes a significant burden on nations, societies, and individuals. Currently, mainstream treatment methods involve mechanical thrombectomy and thrombolysis; however, these often result in poor prognoses. Thus, alleviating the neurofunctional damage caused by ischemic stroke has become an urgent medical challenge.

With the advancement of single‐cell sequencing technology, researchers have gained a deeper understanding of the diverse functions of the different cell types involved in ischemic stroke. Studies have identified microglia as key cells in AIS that play a central role in immune responses. Notably, microglia exhibit multiple distinct subclusters with significant functional differences. Some subclusters participate in inflammatory reactions, whereas others are involved in related metabolic processes, cell differentiation, neural regeneration, exacerbation of AIS‐induced brain injury, and improvement of AIS prognosis. Due to the highly complex immune microenvironment after AIS, it is important to note that basic research and clinical treatments should not aim to universally suppress or activate specific cell types. Instead, it is crucial to identify specific subclusters and selectively regulate harmful subclusters while simultaneously activating beneficial subclusters. Thus, one exploratory avenue is to induce the transformation of detrimental subclusters into advantageous subclusters. In this regard, DHLA‐AuNCs, HMGB1, and DGA have been found to inhibit M1 microglial cell activation or enhance M2 microglial cell activation, thereby inducing neural regeneration.^43^, ^44^ However, it is worth noting that there is currently limited research on the targets involved in the interconversion of different subclusters. At present, the grouping of cell subpopulations in the field of single‐cell sequencing is relatively confusing, which directly affects the horizontal comparison of different research results. Further in‐depth and extensive studies are required to elucidate the regulatory mechanisms underlying this process. In addition, although drugs targeting the mechanisms of ischemic brain injury have been identified in experimental stroke models, clinical trials have not yielded the same positive results as those seen in animal studies.^182^ Clinical translation in the field of ischemic stroke is fraught with difficulties.^183^, ^184^ On the one hand, it may be due to the gap in animal models. The current main experimental animal is rats. Although animal models are of certain value in studying the mechanisms of diseases such as stroke, there are biological differences between animal models and humans, including immune systems, metabolic pathways, genetic background, etc., and there may also be pathophysiological differences and differences in treatment responsiveness. These differences may limit the clinical application of the study results. Future research needs to pay more attention to the differences between animal models and human diseases and actively explore better methods to improve the reliability and predictability of animal models.^185^, ^186^

Single‐cell sequencing technology plays a key role in AIS research. Single‐cell sequencing can not only help identify affected cell types and subtypes and analyze changes in cell heterogeneity after stroke, but can also reveal expression changes in stroke‐related genes, help understand the pathogenesis of stroke, and identify affected genes, providing clues for the development of new targets for stroke treatment. Based on single‐cell sequencing data, personalized treatment strategies can be provided for patients and improve treatment effects. Taken together, single‐cell sequencing technology plays a key role in solving AIS research and can deeply reveal the molecular mechanism of stroke, treatment targets, and the development of personalized treatment strategies.^50^, ^145^, ^178^, ^187^, ^188^ Although single‐cell sequencing technology is now widely used, it is undeniable that single‐cell sequencing technology currently has certain limitations. Single‐cell sequencing technology also presents a number of technical challenges when dealing with single cells. For example, cell capture efficiency, RNA sequencing coverage depth, error rate, etc., may affect the accuracy and reliability of the data. Single‐cell sequencing data may be affected by noise due to intercell differences and experimental errors. In addition, due to the random expression of RNA molecules, the sequencing results of the same cell at different time points or conditions may also be different. Single‐cell sequencing provides an instant snapshot of a cell's state, but cannot capture the dynamic changes in cell state. In the study of the development process, cell subpopulation transformation. In addition, while single‐cell sequencing can identify cell types, in some cases, the boundaries between different cell types can be blurred, leading to uncertainty in classification. Classical bulk RNA sequencing and popular single‐cell sequencing destroy the structural organization of cells and fail to provide spatial information. However, gene expression, or the spatial location of cells in complex tissues, provides key clues to understanding how neighboring genes or cells cross talk and transmit signals. The emergence of spatial transcriptomics has made up for the lack of spatial location information in single‐cell transcriptomics.^189^ Spatial transcriptomics can reveal the spatial distribution of gene expression in brain tissue, which has important implications for understanding the interactions of cell types and complex molecular processes in damaged areas after stroke. Spatial transcriptomics provide spatially resolved gene expression profiles with single‐cell resolution for in‐depth characterization of cell types and states within tissues.^189^, ^190^

## 11 CONCLUSION

In conclusion, with the advent of single‐cell omics, analyzing the mechanism of action of various cells and different subclusters at different time periods holds promise as a research direction to regulate the transformation of harmful subclusters into beneficial clusters, ultimately mitigating brain damage after AIS.

## AUTHOR CONTRIBUTIONS

Yi Huang and Xiang Gao contributed to the conception and design of the study. Xinpeng Deng, Ziliang Hu, Shengjun Zhou, Yiwen Wu, Chenhui Zhou, and Jie Sun organized the database. Xinpeng Deng and Menglin Fu wrote the first draft of the article. Yi Huang reviewed and edited.

## CONFLICT OF INTEREST STATEMENT

The authors in this study declare no conflict of interest.

## Data Availability

Data sharing not applicable to this article as no datasets were generated or analysed during the current study.
